# Happiness and Mental Health in Pre-Operative and Post-Operative Transsexual People

**Published:** 2019-12

**Authors:** Elahe FALLAHTAFTI, Meisam NASEHI, Roya RASULI, Dariush D. FARHUD, Taghi POUREBRAHIM, Hassan ZAREEEIMAHMOODABADI

**Affiliations:** 1. Department of Counseling, Faculty of Educational Sciences and Psychology, Shahid Beheshti University, Tehran, Iran; 2. Department of Biology, Faculty of Basic Sciences and Technologies, Science and Culture University, Tehran, Iran; 3. Department of Counseling, Faculty of Educational Sciences and Psychology, Alzahra University, Tehran, Iran; 4. School of Public Health, Tehran University of Medical Sciences, Tehran, Iran; 5. Department of Psychology, Faculty of Educational Sciences and Psychology, Yazd University, Yazd, Iran

**Keywords:** Happiness, Mental health, Transsexual, Iran

## Abstract

**Background:**

Sex is one of the major social classes in any society. Gender identity as the most fundamental element of human life from beginning to end. While most people with behavior and attitudes appropriate to their physiological gender, but among them are also those sexual behaviors with their physical sex does not match, that say to this group transsexual people. The aim of this study was to investigate happiness and mental health in transsexual individuals before surgery and after surgery.

**Methods:**

This quasi-experimental study, consisted of 42 patients before surgery and after surgery inside Iran in 2016–2017. Snowball sampling method was selected. The instrument was Oxford Happiness Questionnaire (OHQ) and Symptom Check List (SCL25). Data were analyzed using SPSS, *t*-test independent groups and oneway ANOVA.

**Results:**

There was significant difference between transsexual individuals before surgery and after surgery in mean score of happiness (*P*<0.01) (*t*=−4/84). Moreover, there was significant difference between the two groups in mean score of mental health (*P*<0.01) (f=19/13).

**Conclusion:**

Transsexual individuals after surgery experienced more happiness and mental health than those before surgery.

## Introduction

Individuals’ identity is affected by different factors such as ethnicity, nationality, religiosity, profession, and age. However, one of the most significant, critical and deciding factors is identity crisis. The terms of mental-physical gender and identity are interchangeable, but in the current psychology, gender is determined by physiological, biological and anatomical differences genetically determined at birth. Sexual orientation is referred to everything linked with cognitive gender in each society and consequently, the society determines roles, behaviors, preferences and attributes (1). Male or female infant is a biological being not acquired adaptive behaviors of social roles; therefore, the difference between sex and gender should be brought into light (2).

The majority of people have either the characteristics of masculinity or femininity, but some have the characteristics of both men and women (3) called transsexual people. A transsexual individual is someone who suffers from severe identity disorder that is due to the disharmony between mental and spiritual characteristics (4). This contradiction might be obvious in the mind, behavior or social behavior. Transsexual is a term to identify individuals who have inconsistent and contradictory gender identity and do not have the right culture match with their gender identity. In fact, transsexual individual is someone whose mental recognition of self’s gender is different from his sexual organs of birth time (5). Individuals with gender dysphoria generally experience distress resulting from incongruence between assigned and experienced gender (6,7). Causing clinically significant distress or impairment in social, occupational, or other important areas of everyday functioning. People suffering from this disease might pursue hormonal and/or surgical treatments in order to realign their experienced gender with physical appearance (8).

The concept of health is one of the most basic concepts in the complex world of human existence that dates back to the earliest point of history (9). Health is recognized as a human right and a fundamental social objective that is vital for fulfilling the basic needs and improving the quality of human life (10). In fact, health is one of the challenges facing most countries (11). WHO defines mental health as a state of well-being in which the individual has known his ability and has made effective and productive use of them and is useful for his community. Generally, mental health provides for mental hygiene through the prevention of mental illness, factors affecting its outbreak, early diagnosis, prevention of factors that cause the relapse of mental illnesses and creating a healthy environment for proper human relationships (12).

In addition, happiness and vitality as one of the most important human psychological needs occupy the human minds because of the major influence on the formation of human personality and in a word, the human life (13). In recent years, happiness has got of crucial importance because of its role in psychological well-being and social health (14). The scientific interest in individuals wellbeing and its impact on society is focusing on examining the predictors of happiness, exploring what are the benefit of being happy, as well as finding the ways to improve well-being on individual or national levels (15). Happiness is all human beings’ common goal; in a way that all efforts are made to achieve this purpose in life (16). Happiness is the positive value individuals set for themselves (17).

Happiness exerts significant effect on the formation of personality and mental health (18). Goal setting in life is the best predictor of happiness and life satisfaction. Moreover, family and personal goals’ regulation is related to important indices of mental health including life satisfaction, self-esteem and optimism (19).

Happiness and physical health have significant positive relationship with extroversion and conscientiousness. Furthermore, it has negative relationship with neuroticism. Mental health is positively correlated to extroversion, agreeableness and conscientiousness and negatively correlated to neuroticism. Moreover, happiness is positively correlated with mental and physical health. The results of stepwise regression analysis indicate that neuroticism, openness to experience and extroversion predict happiness and physical health. Mental health is predicted by extroversion, conscientiousness, neuroticism and openness to experience. High levels of extroversion and low levels of neuroticism are important predictors of happiness and mental and physical health (20).

There was significant relationship between mental health and happiness (*P*<0.05). Moreover, there existed significant relationship between the lack of anxiety and happiness; lack of depression and happiness (*P*<0.01). Furthermore, there was significant relationship between physical performance, happiness, and happiness. No significant difference was observed between the participants’ happiness in male and female groups (21).

In Netherlands, life quality of transsexual women did not show any significant difference as compared to transsexual men. However, transsexual men showed lower life satisfaction as compared to women. Moreover, transsexual women showed lower physical performance and general health than transsexual men (22). Life satisfaction was low on general health, personal and physical health and role limitations (23). Men did not have a proper understanding of their physical and mental health. The majority of the sample reported that they had active sexual activity before hormone therapy. During hormone therapy and before the surgery, individuals had less tendency for sexual activity. Although, these individuals were able to achieve orgasm during treatment and they all reported that they have more power to reach orgasm (24).

The aim of this study was to investigate happiness and mental health in transsexual individuals before surgery and after surgery.

## Materials and Methods

The sample in the present research comprised transsexual individuals (FTM and MTF) before and after the surgery. Snowball sampling was employed and the transsexual individuals were selected from all over the country due to the limited sample and the sampling was not limited to any provinces. First, one individual was selected by the researcher and s/he was asked for his/her friends who were in the same situation. The sample comprised 66 individuals (42: before the surgery and 24 after the surgery) in 2016–2017.

The individual who was at the pre-surgery stage had completed the necessary steps at the forensic office and had got the permits required for this surgery but had undergone no operation. Moreover, a minimum of one year had passed for the individuals who were at the post-operative stage. Sampling the post-operative individuals was far more difficult because these individuals do not like to get known and try to end their relationships with their friends.

Informed consent was taken from all participants before the study and the study was approved by the local university.

The method used in this research was quasi-experimental. Statistical Society in the present research comprised transsexual individuals (FTM and MTF) before and after the surgery inside Iran. The sample comprised 66 individuals (42: before the surgery and 24 after the surgery). The individual who was at the pre-surgery stage had completed the necessary steps at the forensic office and had got the permits required for this surgery but had undergone no operation. Moreover, a minimum of one year had passed for the individuals who were at the post-operative stage. Sampling the post-operative individuals was far more difficult because these individuals do not like to get known and try to end their relationships with their friends.

Snowball sampling was employed and the trans-sexual individuals were selected from all over the country due to the limited sample and the sampling was not limited to any provinces. First, one individual was selected by the researcher and s/he was asked for his/her friends who were in the same situation.

Investigation of the demographic variables indicated that the mean age of all the transsexual individuals, pre-operative and post-operative individuals were equal to 25.27, 23.76 and 27.92, respectively. No significant between-group difference was observed (*t*=−2.73, *P*=.008). The numbers of pre-operative men and women were 12 and 30, respectively. Whereas, the numbers of post-operative men and women were 15 and 9, respectively. The educational levels were 5, 9, 4, 21 and 3 for pre-operative individuals in the junior high school degree, Diploma, AD, BA, MA or Ph.D., respectively. The educational levels were 0, 4, 1, 15 and 4 for post-operative individuals in the junior high school degree, Diploma, AD, BA, MA or Ph.D., respectively. The results of Chi-square test showed that there is no significant difference between the educational levels of these two groups (χ^2^=5.35).

### Research tools

The Oxford Happiness Questionnaire was derived as an improved version of the Oxford Happiness Inventory by Researchers. The scale has 29 items which include the 20 items of the Oxford Happiness Inventory and an additional 9 items. This questionnaire has 5 scales including life satisfaction, positive mood, health, competency and self-esteem. Responses are based on a 6-point rating scale. Researchers reported acceptable validity for the Oxford Happiness Questionnaire by providing data on correlations with other self-report scales of personality traits, human strengths and subjective well-being. The scale possesses a high scale alpha reliability of 0.91. The inter-item correlations for Oxford Happiness Questionnaire ranged from −0.04 to 0.67 (25). The reliability of this scale was reported to be equal to 0.79 (26). In another research its test-retest reliability was obtained to be equal to 0.78 that showed high content validity (27).

SCL-25 questionnaire is the short form of SCL-90-S. SCL-25 has high correlation with SCL-90-R and is a reliable and valid scale for the assessment of mental pathology. The validity of SCL-25 was assessed by internal consistency and test-retest coefficients. The internal consistency was equal to 0.97 and 0.98 in the female and male groups, respectively. The validity coefficients on a sample of 312 university students in Shahid Chamran University was equal to 0.78 (total sample), 0.77 (female) and 0.79 (male) with time interval of 5 wk. SCL-25 has a correlation of 0.95 with the main questionnaire regardless of the deletion of 65 items (28).

*t*-test for independent groups and one-way variance analysis was used in order to answer the research questions to determine the significant difference between pre-operative and post-operative transsexual individuals in each of the research variables.

## Results

### The inferential findings of the research questions

*t*-test for independent groups and one-way variance analysis was used in order to answer the research questions to determine the significant difference between pre-operative and post-operative transsexual individuals in each of the research variables. However, Shapiro-Wilk and Levene’s test were used to assess the assumptions of normality and homogeneity of variance. The results of Shapiro-Wilk indicated that the assumption of normality and homogeneity of variance exist in the score of happiness and mental health. The results of Levene’s test indicated that there is no significant difference between the variances of the research variables. Therefore, the variance of homogeneity has been observed.

First question: is there any significant difference between the pre-operative and post-operative transsexual individuals’ happiness?

Independent group *t*-test for the significance of mean difference between pre-operative and postoperative transsexual individuals’ happiness indicated that there is significant difference between the mean score of pre-operative and postoperative transsexual individuals’ happiness (*t*= s and one-way variance analysis was used in order to answer 4.84, *P*<.01, Df=64). Therefore, there was significant difference between the happiness of pre-operative and post-operative transsexual individuals.

Second question: is there any significant difference between the pre-operative and postoperative transsexual individuals’ mental health? There existed significant difference between the mean score of pre-operative and post-operative transsexual individuals’ mental health (f=19.13, *P*<.01) (Table 1). In addition, there existed significant difference between the pre-operative and post-operative individuals in the mean score of somatization, anxiety, depression, interpersonal sensitivity, phobia, obsession-compulsion, paranoia and psychosis (f=12.77, *P*<.01), (f=8.14, *P*<.01), (f=12.27, *P*<.01), (f=18.32, *P*<.01), (f=4.16, *P*<.05), (f=11.07, *P*<.01), (f=5.63, *P*<.05) and (f=7.11, *P*≥ .01). Therefore, there was significant difference between the mental health of pre-operative and post-operative transsexual individuals.

**Table 1:** One-way variance analysis (ANOVA) for the significance of mean score difference in pre-operative and post-operative transsexual individuals’ mental health

***Source of change***	***SS***	***Df***	***MS***	***f***	***Level of sig.***
Total score of mental health	4450.98	1	4450.98	19.13	.0001
Somatization	280.51	1	280.51	12.77	.001
Anxiety	72.48	1	72.48	8.14	.006
Depression	44.57	1	44.57	12.27	.001
Interpersonal sensitivity	120.55	1	120.55	18.32	.0001
Phobia	21	1	21	4.16	.04
Obsession-compulsion	98.20	1	98.20	11.07	.001
Paranoia	9.71	1	9.71	5.63	.02
Psychosis	38.28	1	38.28	7.11	.01

## Discussion

Timely diagnosis, treatment and cost of the disease brings about significant psychological distress and negatively affects patients’ quality of life and happiness (29). Income, the heavy burden of debt, a sense of relative poverty as compared with neighbors, access to formal education and physical health are strong predictors of happiness (30). Happy individuals have higher levels of physical and mental health and also life expectation. They attain more occupational and social success (31). Feelings of happiness can be used for curing mental disorders, enhancing hope and as an effort to enhance individuals’ quality of life (32, 33). Happy individuals not only respond more positively and adaptively to the situations and incidences but also have less stress and stronger immune system and are also more creative than depressed individuals. That is to say that happy individuals not only act more successfully in coping with daily stresses but also in stressful and threatening life events (34–36).

Happiness has significant and positive relationship with different aspects of health and a crucial role in the prevention of physical and psychological disorders (37–39). Physical health is a predictor of happiness; therefore, the difference between pre-operative transsexual individuals’ low level of happiness as compared to the postoperative transsexual individuals is significant. Moreover, regarding the previous studies, one of the factors that leads to the lower levels of happiness in pre-operative transsexual individuals as compared to the post-operative transsexual individuals is their worry for the cost and treatment of their disease and this worry is rooted in the family’s rejection as the result of sexual transformation. Family’s lack of support in financial and emotional aspects gives rise to a number of problems. Therefore, transsexual individuals have lower levels of happiness as compared to the normal population.

There is high correlation (0.85) between health and happiness (40, 41). Individuals who introduce happy experiences as the source of their happiness have higher levels of mental health (42). Mental health and the decrease of emotional problems have significant effect on the increase of life satisfaction (43–46). Many of the aspects of the quality of life have significant correlation with mental health. The aspects of physical performance have significant correlation with anxiety, disorder in social performance and depression. Quality of life from the social performance domain has significant relationship with dysfunctional social performance from mental health domain. Anxiety from the scope of mental health was observed more in women as compared to men and the pseudo-physical symptoms was more observed in women as compared to men. Moreover, physical performance from the scope of the quality of life was more significant in men as compared to women and physical pain was more significant in men as compared to women (47).

Patients have low level of mental health and more anxiety, depression and distress. Patients have higher levels of sensitivity in their interpersonal relationships and this is also confirmed among transsexual individuals. These individuals are not satisfied with their physical condition and their lack of satisfaction causes them to get more isolated and dissociable than the normal group. These individuals cannot establish any relationships with others and the fear of getting known as transsexual bothers them and therefore, they prefer to avoid establishing relationship with others.

This is recommended that a group of individuals is investigated to assess the level of happiness and mental health prior to and subsequent to the surgery. Moreover, the factors of gender and sexual satisfaction should also be considered. Furthermore, social relationships, introversion and extraversion of transsexual individuals should also be brought to light.

## Conclusion

There exists significant difference between the mean score of pre-operative and post-operative transsexual individuals’ mental health and happiness

## Ethical considerations

Ethical issues (Including plagiarism, informed consent, misconduct, data fabrication and/or falsification, double publication and/or submission, redundancy, etc.) have been completely observed by the authors.
